# Perioperative Adverse Events in Geriatric Patients: A Comparison of the Predictive Abilities of Three Preoperative Scores

**DOI:** 10.7759/cureus.86805

**Published:** 2025-06-26

**Authors:** Kavita Lacaille, Seetharaman Hariharan

**Affiliations:** 1 Anaesthesia and Intensive Care, The University of the West Indies, St. Augustine, TTO

**Keywords:** geriatric patients, preoperative evaluation, risk prediction, scoring system, surgical outcomes

## Abstract

Introduction

Geriatric patients are increasingly presenting for surgery globally. This study aimed to compare three preoperative scoring systems in their ability to predict perioperative outcomes in geriatric patients who underwent surgical intervention at a tertiary care teaching hospital.

Methods

A retrospective chart review was done to include geriatric patients (65 years and above) undergoing various surgical procedures. American Society of Anesthesiologists (ASA) Physical Status Grades, Revised Lee Cardiac Risk index, and Generic Scores were assigned to all patients. Demographic data and clinical data including preoperative clinical parameters, the procedure, type and duration of anesthesia, surgical specialty, blood loss, intraoperative and postoperative events, length of stay, postoperative events and survival were recorded. The three scores were compared regarding their ability to predict perioperative adverse events.

Results

Fifty-four patients were included in the study. The ages ranged from 65 to 87 years (Mean 73.5, 5.8 SD). Females were the majority of patients (64.8%). Patients belonged to general surgery, urology, and thoracic surgery. About 7.4% of the patients developed postoperative events requiring ICU admission. Within the geriatric range, age was not a significant factor impacting perioperative adverse events. Duration of surgery, anesthesia, and hospital length of stay were significantly higher in patients who had perioperative adverse events. With respect to discriminant ability for predicting perioperative adverse events, the area under the Receiver Operating Curve for Lee Cardiac Index was 0.85, 0.67 for the Generic Score, and 0.62 for ASA Physical Status Grades.

Conclusion

With the limitation of a small sample size, within the patients studied, the revised Lee Cardiac Risk Index was able to predict immediate perioperative adverse events better when compared to the ASA Physical Status Grades and a Generic Score in geriatric patients.

## Introduction

Patients undergoing surgical procedures are at a small risk of experiencing critical incidents pertaining to anesthesia and surgical errors [1]. Beyond this unanticipated risk, perioperative morbidity and mortality can be influenced by acute physiological derangements, the existence of co-morbidities, the extent of end-organ damage, the type of surgical intervention, and the type of anesthesia. It is common knowledge that geriatric patients are much more vulnerable to perioperative adverse outcomes when compared to younger age groups. An adverse perioperative outcome usually delays recovery and lengthens hospital stay. Commonly encountered postoperative complications in elderly patients may include wound infection/dehiscence and respiratory failure secondary to pneumonia and atelectasis. Other adverse outcomes, such as acute myocardial infarction, cerebrovascular accidents, and cognitive decline in the elderly, may also cause a long-term deterioration in a patient’s baseline physiological status [2].

Preoperative risk assessment of a patient, when conducted by a member of the anesthesia team, not only serves to enhance patient rapport but also to assess the patient in a comprehensive manner to determine if further physiological optimization is required before anesthesia and surgery [3]. It also serves to predict which patients are at a higher risk of developing perioperative complications [3]. Preoperative assessments should also aim to quantify the risk involved so that patients can be stratified based on this quantification [1]. This stratification will allow informed decision-making regarding perioperative care, and the overall patient outcome and prognosis.

When risk assessments suggest that the patient’s overall outcome may be poor and the likelihood of an adverse outcome is unavoidable, this can assist in planning the treatment options [1]. This is especially true with geriatric patients who present for surgical intervention, where the risk of an adverse outcome may significantly outweigh the benefit of surgery. Geriatric patients have a wide range of ‘physiological ages’ that may not necessarily correlate with their chronological age. Hence, ideally, their preoperative assessment should be multidisciplinary [2].

Any tool for risk prediction must be easy to perform, reasonably quick to institute, objective in its assessment, reliable, and easily reproducible in all patients without significant inter-observer variability [4]. The American Society of Anesthesiologists (ASA) Physical Status Classification system and the Revised Lee Cardiac Risk Index for cardiovascular risk are very commonly applied risk assessment tools [1,4]. They have the advantage of exclusively utilizing the preoperative clinical parameters. The ASA Physical Status Classification is a subjective scoring system that aims to predict postoperative morbidity and mortality [5]. It involves a total of six Grades (I to VI) allocated based on the presence of a patient’s co-morbidities and overall effect on functional status.

An additional subscript E connotes a patient presenting for an emergency operation [5]. The allocation of the ASA Grades can be very subjective in nature and subject to much inter-observer variability [6,7]. For example, the definition of controlled versus uncontrolled co-morbidities is subject to significant variability as it is dependent on the opinion of an anesthesiologist [8]. The same is true with the interpretation of co-morbidities that may be considered a ‘constant threat to life’. The effect of chronological age of a patient on the allocation of the ASA Grade is also subject to much variability [6,8]. The classification suggests that extremes of age could increase a patient’s ASA Grade, but does not specify which specific numerical age should be the threshold to increase the score. Although the reliability of the ASA classification in the accurate prediction of morbidity and mortality may be of concern due to its interpreter variability, it is still the most commonly used preoperative assessment tool by anesthesiologists. The Revised Lee Cardiac Risk Index assigns a cardiovascular risk depending on a patient’s clinical parameters.

A percentage risk is assigned based on the number of positive predictors present with, 0 predictors amounting to a 0.4% risk of cardiovascular complication, 1 predictor a 0.9% risk, 2 predictors a 6.6% risk, and 3 or more predictors a greater than 11 % risk [1]. It is quick and easy to perform, objective in assessment, and easily reproducible without significant inter-observer variability. However, it focuses only on the prediction of perioperative cardiovascular risk [9]. While a cardiovascular event is a significant contributor to perioperative morbidity and mortality, it may not be the only factor.

It is obvious that the presence of comorbidities, decreased renal and cardiac reserve, anemia, and cognitive dysfunction significantly increases the perioperative morbidity and mortality in the elderly population. However, no preoperative scoring system currently exists that incorporates an assessment of all these individual parameters. With this consideration, a Generic Score was formulated de novo, based on the presence of co-morbid conditions such as diabetes mellitus, hypertension, ischemic heart disease, anemia, cognitive dysfunction, and renal dysfunction. These parameters were chosen from published literature, wherein evidence suggested that they are associated with a higher risk of perioperative morbidity and mortality in the elderly. This Generic Score is quick and easy to perform, relatively objective in assessment, and may allow for the prediction of the overall risk of morbidity and mortality. With this overall background, the present study tested the ability of these three scoring systems to predict the overall perioperative risk in geriatric surgical patients.

## Materials and methods

This study was conducted as a retrospective chart review after obtaining approval from the Campus Research Ethics Committee of the University of the West Indies, St. Augustine. The setting where the study was conducted was a tertiary healthcare facility in Trinidad and Tobago and included patients who had a surgical intervention performed within one calendar year. Only clinical and demographic data necessary for the study was extracted from the medical record files of patients. No personally identifying information was extracted from the charts; data collection forms were labelled by a numeric code assigned to maintain confidentiality. Data collection forms were also not accessible to anyone who was not associated with this research. Data analyses were performed and stored on a password-protected computer. Informed consent from individual patients was waived due to the design of the study (chart review).

Inclusion criteria were a chronological age of 65 years or older, having undergone a surgical intervention under anesthesia. Sedation with monitored anesthesia care, regional anesthesia, general anesthesia, and combined regional/general anesthesia were all included. A documented preoperative anesthetic assessment was considered essential for inclusion in the sample. This assessment must have been performed by anesthesia personnel and clearly documented in each patient's medical record for review.

Patients were excluded from the sample if they were under 65 years of age or if their surgical intervention occurred outside the specified calendar year of the study. Surgical interventions carried out exclusively under local anesthesia provided by a member of the surgical team (without an anesthesiologist) were excluded. Patients who underwent cardiac and neurosurgical interventions were also excluded due to many confounding variables, which may lead to bias.

Daily surgical listings, daily manager’s reports for operative procedures, surgical logbooks, and the postoperative care unit logbooks stored within the main operating theatres were reviewed. Data recorded included patient demographics such as patient’s chronological age, gender, presence of co-morbidities, preoperative serum hemoglobin, and preoperative serum creatinine level. It also included the type, duration, and estimated blood loss for the patient’s given surgical intervention and the type and duration of anesthesia administered. An ASA Physical Status Grade, a Revised Lee Cardiac Risk index score and Generic Score were assigned to each patient based on the data extracted. Table 1 depicts the new Generic Score. Tables 2, 3 depict the ASA scoring system and the Revised Lee Cardiac Risk Index, respectively.

**Table 1:** Generic score IHD: Ischemic Heart Disease, Cr: Serum Creatinine, Hb: Hemoglobin.

Parameter	0 points	1 point	2 points	3 points
Diabetes Mellitus	Nil	Controlled	Uncontrolled	Chronic insulin use
Hypertension	Nil	Grade 1	Grade 2	Grade 3
IHD	Nil	Class 1	Class 2	Class 3 or 4
Renal Dysfunction	Nil	Acute (Cr 1.7-2 mg/dL)	Acute (Cr > 2mg/dL)	Chronic Renal Failure
Anemia	Nil	Hb 8-10 g/dL	Hb 6-8 g/dL	Hb<6 g/dL
Cognitive dysfunction	Nil	Acute	Chronic	-

**Table 2:** ASA scoring system ASA: American Society of Anesthesiologists.

Score	Description
ASA I	No co-morbidities
ASA II	Presence of co-morbidities that are well-controlled
ASA III	Co-morbidities that are not well-controlled but not a constant threat to life
ASA IV	Co-morbidities that are not well-controlled and a constant threat to life
ASA V	Moribund patient
ASA VI	Brain-dead patient

**Table 3:** Revised Lee Cardiac Risk Index

Lee Score	Risk Class	Percentage of Risk
0	1	0.4
1	2	0.9
2	3	6.6
3 or more	4	11

Postoperative data, including the presence of any perioperative event that may have contributed to morbidity and mortality, and the length of hospital stay, were also recorded from each patient’s medical record file. All postoperative complications were documented for individual patients. These included myocardial infarction, pulmonary embolism, deep vein thrombosis, pulmonary edema, cerebrovascular accident, prolonged mechanical ventilation, intensive care unit admission, infection, wound dehiscence, new onset dysrhythmia, new onset cognitive dysfunction, and in-hospital mortality (if any). Evidence of a prolonged hospital stay or the presence of a postoperative complication as listed above was considered a proxy for morbidity.

Data collected were entered into Microsoft Excel and Statistical Program for Social Sciences (SPSS) Software, version 14 (IBM Corp., Armonk, NY). Descriptive analyses were performed. Inferential analyses included Fisher’s Exact test to compare the postoperative adverse events between different categories of patients, independent t-tests to compare variables between patients who experienced postoperative adverse events and discriminant analyses using Receiver Operating Characteristic (ROC) curve analyses to compare the predictive ability of the scoring systems. Statistical significance was considered at the level of p<0.05.

## Results

The total number of patients aged 65 years and older who underwent surgical intervention during the calendar year of study was 150. However, the medical record files of only 54 patients had the complete information needed for the study, and hence, 54 patients were enrolled for analyses. There were 35 female patients (64.8%) and 19 male patients (35.2%). The age of the patients ranged from 65 to 87 years (Mean 73.5 years, Standard Deviation (SD): 5.78). Thirty-five patients (64.8%) suffered a chronic medical condition, while 19 patients (35.2%) denied any history of chronic disease. Of all the co-morbidities, hypertension was the most prevalent medical illness in 29 patients (53.7%), followed by diabetes mellitus in 15 (27.8%) patients. In total, 22 patients (40.7%) suffered from more than one medical problem. The combination of diabetes mellitus and hypertension was the most prevalent (22.2%) among patients who had more than one co-morbidity. Ischemic heart disease, chronic kidney disease, asthma, atrial fibrillation, cancer, Alzheimer’s disease, deep vein thrombosis, cerebrovascular disease, myasthenia gravis, and sarcoidosis were the other comorbidities reported. Five patients admitted had a history of current cigarette smoking, two patients had a history of alcohol consumption, and one patient was a marijuana user.

Forty-eight patients (89%) had a preoperative hemoglobin more than 10 g/dL, four patients had values in the range of 8-9.9, and one patient each in the range of 6-7.9 and less than 6, respectively. The serum creatinine level was normal in 51 (94.4%) patients and more than 2 mg/dL in three patients. Three patients (5.6%) also had preoperative cognitive dysfunction. The majority of the patients (46, 85.2%) had elective surgical interventions, and eight (14.8%) had emergency surgeries. General Surgery was the most common specialty in both emergency and elective surgeries, accounting for 72% of elective and 50% of emergency surgical procedures, respectively. Table 4 shows the distribution of patients according to various specialties.

**Table 4:** Characteristics of the surgical cases

Specialty	Surgery Urgency (%)		Postoperative Event (%)	
	Elective	Emergency	No	Yes
General Surgery	23.0	14.8	50.0	7.4
Thoracic Surgery	42.6	0	16.7	0
Urology	24.0	0	24.0	0
Ophthalmology	1.9	0	1.9	0
Total	85.2	14.8	92.6	7.4

Exploratory laparotomies, inguinal hernia repairs, and mastectomies were the commonest operations performed (13% each). JJ stents, video-assisted thoracoscopy, and cholecystectomy were other common procedures. Fifty-two (96.3%) of them had general anesthesia, one patient had a combined general/ regional anesthesia, and one had sedation/ monitored anesthesia care (MAC). The surgical duration ranged from 5 to 662 minutes (Median 66.5 min; IQR: 39.25, 101.5). The anesthesia duration ranged from 5 minutes to 240 minutes (Median 90 min; IQR: 50, 120 min). The hospital length of stay ranged from 1 to 29 days (Median 2 days; IQR: 1, 4.25).

**Figure 1:**
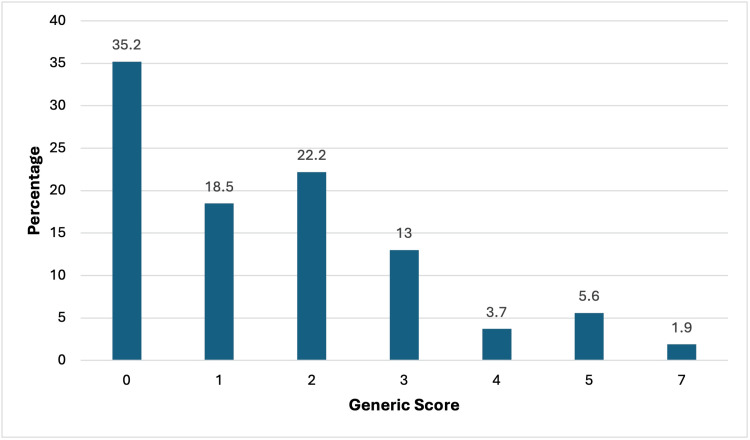
Distribution of patients by Generic Score

There were 15 patients (35.2%) who were assigned to ASA physical status Grade I and 37 (61.1%) with ASA Physical Status Grade II. There was one patient in each ASA III and IV grade. The Revised Lee cardiac risk index classified 30 patients with a Class 1 risk of a perioperative cardiovascular event. A further 17 patients (27.8%) were classified as having a Class 2 risk of developing a perioperative cardiovascular event, and six patients Class 3 and one patient Class 4 risk. With respect to the Generic Score, most patients had lower scores; the distribution is shown in Figure 1.

There were only four cases (7.4%) with a recorded occurrence of a postoperative event considered to have increased their postoperative morbidity. All four patients were carded for emergency surgery and developed multiple perioperative complications. All four required postoperative ICU admission and mechanical ventilation. Cerebrovascular event, pulmonary embolism, Acute Kidney Injury, new-onset dysrhythmia, and new-onset cognitive dysfunction were the complications recorded. There was no perioperative mortality recorded within this study during the patients' stay in the hospital. However, it could not be ascertained whether death occurred after discharge from the hospital. Table 5 shows the distribution of patients according to the three different scoring systems and according to the presence of postoperative adverse events.

**Table 5:** Distribution of the scores in patients

Scoring system	Postoperative Complications		Total
ASA	No	Yes	
I	14	1	15
II	35	2	37
III	1	0	1
IV	0	1	1
Lee Cardiac Risk Index			
Class 1	30	0	30
Class 2	15	2	17
Class 3	4	2	6
Class 4	1	0	1
Generic Score			
0	18	1	19
1	10	0	10
2	11	1	12
3	6	1	7
4	2	0	2
5	3	0	3
7	0	1	1

A Fisher's Exact test revealed a statistically significant association between the Revised Lee Cardiac Risk index and the occurrence of perioperative complications (p=0.016). There was no statistically significant association between perioperative complications and ASA Physical Status Grade or Generic Score. Independent t-tests were performed for comparison of variables with the occurrence of perioperative adverse events, as shown in Table 6.

**Table 6:** Comparison of variables according to postoperative events p-value by Independent t-test

Variable [Mean (SD)]	Postoperative adverse event		p-value
	No (n=50)	Yes (n=4)	
Age (y)	73.30 (5.93)	76.00 (2.58)	0.37
Preoperative hemoglobin (g/dL)	12.26 (1.69)	9.98 (4.94)	0.03
Surgery Duration (min)	77.68 (92.81)	212.50 (17.08)	<0.001
Anesthesia Duration (min)	83.66 (42.88)	232.50 (15.00)	<0.001
Length of Stay (d)	2.98 (3.34)	18.00 (8.37)	<0.001

**Figure 2:**
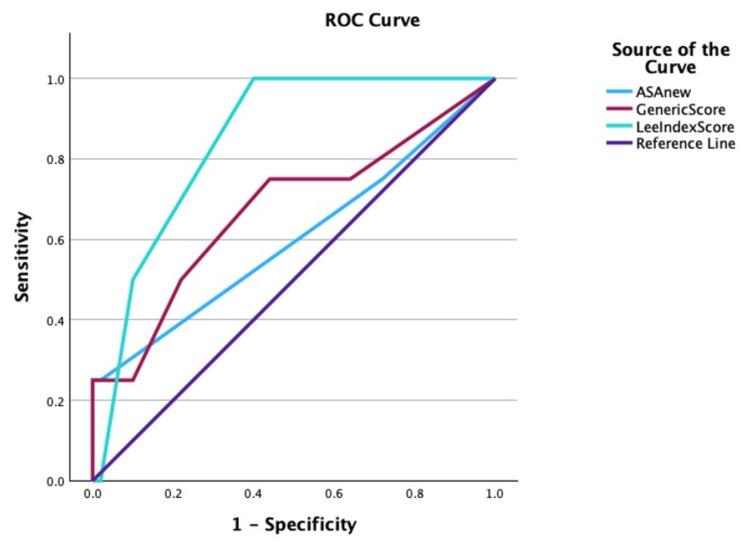
Receiver operating characteristic curves for the discriminant ability of the three scoring systems

Age was not significantly different between those who developed adverse events and those who did not. Preoperative hemoglobin was lower (mean 9.98 g/dL) in patients with perioperative adverse events, compared to those who did not have complications (mean 12.26 g/dL) (p=0.03). Duration of anesthesia and surgery was significantly longer in patients who developed adverse events compared to patients who did not have these events (p<0.001). Similarly, the hospital length of stay was also significantly longer in the group with postoperative adverse events (p<0.001). A Receiver Operating Characteristic (ROC) curve analysis was done to test the discriminant ability of all the scoring systems (Figure 2). When considering area under the curves (AUC), the Revised Lee Cardiac Risk index had a better discriminant ability of perioperative complications with an AUC of 0.84, than the ASA Physical Status Grade and the Generic Score. The ASA Physical Status Grade had an AUC of 0.62, compared to the Generic Score with an AUC of 0.67.

Table 7 shows the ROC analysis details.

**Table 7:** Receiver operating characteristic curve analyses for the three scoring systems A p-value is generated under the assumption that the area under the curve of 0.5 represents the null-hypothesis.

Scoring system	Area Under the Curve	Std. Error	p- value	95% Confidence Interval	
				Lower bound	Upper Bound
Revised Lee Cardiac Risk Index	0.845	0.070	0.023	0.708	0.982
ASA Physical Status Grades	0.618	0.174	0.509	0.260	0.940
Generic Score	0.673	0.161	0.254	0.357	0.988

## Discussion

This study evaluated the ability of three different risk assessment tools in predicting immediate perioperative adverse events in geriatric patients undergoing surgery in a tertiary care teaching hospital. The Revised Lee Cardiac Index performed better than the ASA Grade and a newly formulated Generic Score.

In the present study, an adverse event was recorded by the presence of one or more of the following perioperative complications: myocardial infarction, pulmonary oedema, pulmonary embolism, deep vein thrombosis, postoperative mechanical ventilation, ICU admission, new onset cognitive dysfunction, new onset cardiac dysrhythmia, and death. Minto and Biccard suggested that postoperative complications are commonly under-reported [1]. They postulated that many pathophysiological derangements, which are not routinely recognized or measured, may be the precursors of postoperative complications. For example, intraoperative myocardial ischemia may go unrecognized (and hence unreported) unless it progresses to either myocardial infarction or clinically overt manifestations such as hemodynamic instability. Such silent intraoperative ischemia may consequently lead to other organ dysfunctions at a much later date. In the present study, no intraoperative adverse events were recorded for any patient, but it is entirely possible that unrecognized events could have occurred, which would have manifested later. Minto and Biccard also suggested that the true prevalence of postoperative complications was likely to be underestimated due to the lack of long-term follow-up beyond the postoperative period [1]. As such, late complications attributable to surgical interventions are often missed in the elderly. The current study followed patients only until the perioperative period of the stay in the hospital, and it is likely that late complications might have been missed.

Any tool to predict the development of postoperative adverse events is always fraught with inconsistencies and poor performance due to the multiple factors involved, including the care offered and the varied case-mix in different settings. Tomoaki and Yoshihisa suggest that ASA Physical Status Grade II must be of a broader range than proposed initially [10]. Age has never been incorporated as a factor into the ASA Physical Status classification. Despite this, many anesthesiologists may automatically assign Grade II for geriatric patients regardless of comorbid illnesses. This can be controversial since ageing cannot be considered as a ‘disease’ state per se. In the present study, if patients were assigned an ASA Physical Status Grade II (based on chronological age alone), documented perioperative adverse events were very minimal within this category. A previous report has suggested that otherwise healthy elderly patients do not have an increased postoperative complication rate when compared to younger patients [11]. This may suggest that automatically upscaling ASA Physical Status Grades exclusively based on chronological age does not significantly alter the predictive capability of the ASA Physical Status grading. Chronological age alone is therefore unlikely to be an independent predictor of increased postoperative morbidity and mortality. Many previous studies have shown that age on its own is a poor predictor of mortality, morbidity, or length of stay in hospital [12-14].

Among those who had postoperative complications in the present study, 50% were of ASA physical status Grade I, and 25% were ASA physical status Grade II. This may also suggest that in 50% of this group, the occurrence of a postoperative complication was likely associated with a factor other than chronological age and comorbidity. These patients were, in fact, undergoing emergency exploratory laparotomy for intra-abdominal pathology. It is likely that these patients had pathophysiological changes secondary to intra-abdominal pathology that contributed to the development of a post-operative complication. Emergency surgical intervention has been shown to increase the risk of developing postoperative complications [2].

McNicol et al. found that thoracic surgical intervention had an increased mortality and morbidity [15]. However, in the current study, patients who underwent thoracic surgical intervention experienced no adverse events. These cases were all elective in nature, which may suggest that they were optimized as best as possible to avoid an increased postoperative morbidity and mortality. In the study performed by McNicol et al., looking at postoperative complications and mortality in older patients having non-cardiac surgery, the prevalence of comorbidities was 70% [15]. This is comparable to the finding of the present study, where the prevalence of co-morbidity was 64.8%. However, McNicol et al. demonstrated a significantly higher incidence (19%) of postoperative complications. In contrast, only 7.4% of patients in the current study developed postoperative complications. Mc Nicol et al. also demonstrated a much higher rate of postoperative intensive care admission (20%) than that of the current study (7.4%). This lower complication rate may have been attributable to the sample selection, wherein a significantly larger number of patients who underwent elective surgical intervention (85.2%). A high proportion of patients had lower risk predictive scores.

In a study by Howes et al., the mean length of stay was 20.4 days in the elderly [16]. In the current study, it was 18 days for those with an adverse outcome. This suggests that once a complication occurs in a geriatric patient, they require a longer period for recovery, probably due to the decrease in physiological reserve associated with ageing. Surgical operative duration has been shown to be an independent risk factor for longer hospital stay, with longer durations leading to increased length of stay [17]. In the present study, in patients with adverse outcomes, anesthesia and surgical duration, as well as the length of stay, were significantly higher.

The ASA Physical Status classification was not demonstrated to be a good predictor of postoperative morbidity and mortality in this study. This is in contrast to a previous study, which suggests that ASA physical status classification is a good predictor of postoperative mortality and morbidity despite its subjective nature and problems with inter-observer variability [13]. However, ASA Physical Status Classification has always been a matter of dispute within the geriatric population [18,19]. In the present study, the number of patients studied and who indeed had postoperative morbidity was small. It is possible that a larger, more diverse sample with a greater incidence of postoperative complications is needed to prove an association and validate its predictive ability. The Generic Score also did not prove to be a good predictor of increased postoperative complications despite including known parameters that contribute to increased perioperative morbidity and mortality. Again, the sample size was too small to demonstrate its validity.

Stonelake et al. indicated that the Revised Lee Cardiac Risk index underestimates the risk when overall morbidity and mortality are considered [20]. However, in the present study, the Revised Lee Cardiac Risk index was shown to be a better predictor of overall morbidity compared to other scores. Surprisingly, the Revised Lee Cardiac Risk Index, designed to estimate cardiac-related complications only and not the overall risk, proved to be the better predictor in the present study.

The retrospective design of the study, lack of long-term follow-up of patients for late postoperative complications, lack of a proper electronic database for individuals undergoing surgical interventions, and an overall small sample size, reducing the power of the study, are the major limitations of the study. Due to the small sample size, the results cannot be generalized. Frailty is also considered to be a significant predictor of perioperative morbidity and mortality in the geriatric population. It was not possible to include a measurement of frailty as a component of the Generic Score, as frailty is not commonly assessed in the study institution. It is possible that the predictive ability of the Generic Score might have improved by the addition of a frailty assessment.

## Conclusions

Despite the limitation of a small sample size, in conclusion, the present study evaluated the prognostic ability of three scoring systems to predict the perioperative morbidity and mortality in geriatric surgical patients. A 'generic' score was devised *de novo* for this research based on the common parameters encountered in older patients in the study institution, and was tested for its validity. This score was compared with two existing scoring systems, namely the Revised Lee Cardiac Index and the traditional American Society of Anesthesiologists (ASA) Physical Status Grading system. The study found that the Revised Lee Cardiac Risk index predicted perioperative adverse events in geriatric surgical patients better among the three studied scoring systems. The 'generic' score did perform better than the ASA Physical Status Grades. The generic score can be adapted, modified, and tested in a larger sample to improve its validity.
